# Intensive Systolic Blood Pressure Lowering and Kidney Disease Progression in IgA Nephropathy: A Cohort Study

**DOI:** 10.3389/fmed.2022.813603

**Published:** 2022-02-16

**Authors:** Guizhen Yu, Jun Cheng, Yan Jiang, Heng Li, Xiayu Li, Jianghua Chen

**Affiliations:** ^1^Kidney Disease Center, First Affiliated Hospital, College of Medicine, Zhejiang University, Hangzhou, China; ^2^Key Laboratory of Kidney Disease Preventsion and Control Technology, Hangzhou, China; ^3^National Key Clinical Department of Kidney Diseases, Hangzhou, China; ^4^Institute of Nephrology, Zhejiang University, Hangzhou, China; ^5^Third Grade Laboratory Under the National State, Administration of Traditional Chinese Medicine, Hangzhou, China

**Keywords:** IgA nephropathy, kidney failure, systolic blood pressure, proteinuria, renoprotective

## Abstract

**Background:**

Hypertension has been shown to be an important risk factor in IgA nephropathy (IgAN). The 2021 the Kidney Disease Improving Global Outcomes (KDIGO) Guideline proposes a target systolic blood pressure (SBP) of less than 120 mmHg in patients with Chronic Kidney Disease (CKD) not receiving dialysis. However, whether lowering SBP from <140– <120 mm Hg is renoprotective is unknown. This study aims to evaluate the association of SBP and the progression of IgAN, then explore whether lowering SBP from <140– <120 mm Hg is renoprotective.

**Methods:**

Overall, 2,240 patients with IgAN were enrolled in this study. Cox proportional hazards models and restricted cubic splines were used to estimate the associations between SBP and kidney failure events which are defined as 50% estimated glomerular filtration rate (eGFR) decline or kidney failure.

**Results:**

After a median follow-up of 30.05 months, 217 (9.69%) patients reached composite kidney failure events. The association of SBP and kidney failure events showed a linear relationship. The risk of kidney failure events was greater with higher SBP. Compared with SBP <120 mm Hg, the hazard ratio was 1.85 (1.16–2.97, *p* = 0.010) for SBP <140 mm Hg after adjustment for traditional risk factors. The renoprotective benefits of therapy targeting SBP <120 mm Hg from SBP <140 mm Hg was detectable within the subgroup with proteinuria >1.0 g/d, CKD 1-3a stage, but not those with proteinuria ≤ 1.0 g/d and CKD 3b-4 stage.

**Conclusions:**

In patients with IgAN, SBP was independently associated with composite kidney failure events. Lowering SBP from <140– <120 mm Hg was renoprotective.

## Introduction

Immunoglobulin A nephropathy is one of the most common primary glomerular diseases worldwide, approximately 20–40% of patients progressed to end-stage kidney disease (ESKD) in 20 years (1–4). IgA nephropathy is characterized by a highly variable clinical course ranging from a benign incidental condition to rapidly progressive kidney failure (5). In addition to persistent proteinuria and hematuria, decreased glomerular filtration rate, and at the onset of biopsy, hypertension is an important risk factor of progression to kidney failure in patients with IgA nephropathy (6–12).

The Kidney Disease: Improving Global Outcomes (KDIGO) guidelines suggested a systolic blood pressure (SBP) target of <120 mm Hg for adults with high blood pressure (BP) and chronic kidney disease (CKD) because of the cardiovascular and survival benefits rather than renal benefits (13, 14). In earlier randomized control trial (RCT) studies, more vs. less intensive BP lowering did not demonstrate a benefit of lowering BP targets on kidney failure events in CKD populations (15–17). However, some smaller trials have suggested that lowering the SBP reduces the rates of CKD progression among patients with greater proteinuria (18–20). Whether lowering the SBP from <140– <120 mm Hg is renoprotective is uncertain, especially in those with age <50 years, proteinuria >1 g/d, and advanced CKD (13). IgA nephropathy is the main cause of CKD, therefore, here we aim to examine the effect of SBP on renal survival, then detect the renoprotective benefits of targeting SBP <120 mm Hg from SBP <140 mm Hg.

## Methods

### Study Population

A total of 2,240 patients with IgA nephropathy diagnosed between 2002 and 2019 in the First Affiliated Hospital, Zhejiang University School of Medicine were selected. Patients mainly come from Southeast China. Secondary IgA nephropathy such as IgA vasculitis, rheumatic disease, and systemic lupus was excluded.

All participants provided written informed consent for using pathological and clinical data, this study was approved by local ethics committees.

### Data Collection and Pathologic Manifestation

In our study, both arms were used for BP measurement. Before measurement, the patient sat in a chair relaxing for >5 min, the patient could not speak during the measurement, the arm which gives the higher reading was used for subsequent readings. Repeated measurements were made after 1–2 min, the average of ≥2 readings obtained was used as the patient's BP.

Clinical data including age, sex, serum creatine level, 24-h urine protein excretion, systolic/diastolic blood pressure at the time of kidney biopsy, and the use of antihypertension medications (including ACEis, ARBs, and calcium-channel blockers) were collected from the medical records. Pathologic elements were evaluated according to the Oxford classification (21, 22). The eGFR was calculated using the Chronic Kidney Disease Epidemiology Collaboration (CKD-EPI) equation (23). Mean arterial pressure (MAP) was defined as the diastolic pressure plus one-third of the pulse pressure. The composite kidney failure event was defined as 50% eGFR decline or end-stage kidney disease (ESKD). ESKD was defined as eGFR <15 ml/min per 1.73 m^2^ or the need for kidney replacement therapy (KRT) (including peritoneal dialysis, hemodialysis, or kidney transplantation). In order to assess the graded effect of SBP on renal survival, SBP was divided into three levels (<120 mm Hg, 120–139 mm Hg, and ≥140 mm Hg). Participants were also divided into two groups according to age (<50 years and ≥ 50years), baseline proteinuria (>1 g/d and ≤ 1g/d), and CKD stage (G1-3a stage and G3b-4 stage).

### Statistical Analyses

Categorical data were expressed as proportion and frequency and were compared using the chi-squared test. Continuous variables were presented as mean ± standard deviation and were assessed with a *t*-test or ANOVA as required for normally distributed data and nonnormally distributed data were compared using the Mann–Whitney test. Restricted cubic splines were used to determine the association of SBP levels with composite kidney failure events, three knots were placed at the 25th, 50th, and 75th for the cubic splines. The relationship between parameters and renal survival was estimated using unadjusted and multivariable-adjusted Cox models. The traditional risk factors of IgA nephropathy including sex, age, proteinuria, eGFR, and Oxford classification scores were adopted in multivariable-adjusted Cox models. Kaplan-Meier analysis was used to derive the cumulative kidney survival curves, the difference between the curves was analyzed using a log-rank test. Data were analyzed using SPSS software version 24.0 and Stata software, version 14.0 (SPSS, Chicago, IL). A two-sided *p* < 0.05 was considered statistically significant.

## Results

### Clinical Data

Clinical data are summarized in Table 1. Among 2,240 patients with IgA nephropathy, there were 1,160 (51.79%) men with a mean age of 38.36 ± 12.37 years. At the time of renal biopsy, the mean SBP, diastolic BP (DBP), and Mean arterial pressure (MAP) were 126.17 ± 18.22, 79.80 ± 13.63, and 95.26 ± 14.27 mm Hg respectively. The initial proteinuria was 0.94 (0.47, 1.78) g/d, the eGFR was 84.04 ± 29.34 ml/min per 1.73 m^2^. During a mean follow-up of 30.05 (14.14, 58.98) months, 217 (9.69%) cases developed composite kidney failure events, including 194 (8.66%) with 50% eGFR decline events and 163 (7.28%) with ESKD events.

**Table 1:** The characteristics of IgA nephropathy patients at the onset of renal biopsy.

**Characteristics**	**Total (*N* = 2,240)**	**SBP (mm Hg)**			
		** <120 (Group 1, *n* = 928)**	**120 to 139 (Group 2, *n* = 821)**	**≥140 (Group 3, *n* = 491)**	***P* value**
Baseline					
Sex (% male)	1,160 (51.79)	574 (61.85)	367 (44.70)	219 (44.60)	<0.001
Age (year)	38.36 ± 12.37	36.59 ± 11.66	38.46 ± 12.47	41.54 ± 12.85	<0.001
SBP (mm Hg)	126.17 ± 18.22	109.75 ± 8.00	129.34 ± 5.25	151.91 ± 12.58	<0.001
DBP (mm Hg)	79.80 ± 13.63	70.21 ± 9.26	82.27 ± 9.07	93.80 ± 12.97	<0.001
MAP (mm Hg)	95.26 ± 14.27	83.39 ± 8.07	97.96 ± 6.81	113.17 ± 11.39	<0.001
Proteinuria (g/d)	0.94 (0.47, 1.78)	0.81 (0.43, 1.46)	0.94 (0.46,1.77)	1.28 (0.66, 2.45)	<0.001
eGFR (ml/min per 1.73 m^2^)	84.04 ± 29.34	91.39 ± 28.16	81.74 ± 28.51	73.94 ± 29.33	<0.001
Oxford classification, no. (%)					
M1	413 (18.44)	121 (14.63)	188 (19.26)	104 (23.80)	<0.001
E1	217 (9.69)	83 (10.04)	83 (8.50)	51 (11.67)	0.162
S1	1,533 (68.44)	570 (68.92)	669 (68.55)	294 (67.28)	0.832
T1-T2	237 (10.58)	52 (6.29)	109 (11.17)	76 (17.39)	<0.001
C1-C2	1108 (49.46)	448 (54.17)	456 (46.72)	204 (46.68)	0.003
CKD stages, no. (%)					
1	1042 (46.52)	528 (56.90)	346 (42.14)	168 (34.22)	<0.001
2	653 (29.15)	249 (26.83)	267 (32.52)	137 (22.41)	0.026
3	476 (21.25)	139 (14.98)	184 (22.41)	153 (31.16)	<0.001
4	69 (3.08)	12 (1.29)	24 (2.92)	33 (6.72)	<0.001
Follow-up and outcome					
Follow-up interval (months)	30.05 (14.14, 58.98)	29.23 (14.42, 56.20)	28.00 (13.43, 51.57)	29.57 (13.18, 56.95)	0.569
Treatment with antihypertension agents	1,659 (74.06)	600 (64.66)	628 (76.49)	431 (87.78)	<0.001
50% eGFR decline, no. (%)	194 (8.66)	42 (4.53)	77 (9.38)	75 (15.27)	<0.001
ESKD, no. (%)	163 (7.28)	29 (3.13)	63 (7.67)	71 (14.46)	<0.001
Composite kidney failure events, no. (%)	217 (9.69)	46 (4.96)	84 (10.23)	87 (17.72)	<0.001

*This cohort included 2,440 patients with IgA nephropathy, including 173 cases without baseline proteinuria data. The composite kidney failure event was defined as 50% eGFR decline or ESKD*.

*SBP, systolic blood pressure; DBP, diastolic blood pressure; MAP, mean arterial pressure; M1, mesangial hypercellularity; E1, endocapillary hypercellularity; S, segmental glomerulosclerosis/adhesion; T, severity of tubular atrophy/interstitial fibrosis. C, presence of crescent*.

The patients were divided into three groups according to the SBP levels as shown in Table 1. There were 928 cases with SBP <120 mm Hg (Group 1), 821 cases with 120–139 mm Hg (Group 2), and 491 cases with SBP≥140 mm Hg (Group 3). With higher levels of SBP, the incidence rate of composite kidney failure events was higher; similar results were observed when the initial proteinuria and eGFR were analyzed. Patients with higher SBP showed a higher prevalence of Oxford M1 and T1-2 lesions and lower Oxford C lesions, there was no significant difference in the prevalence of Oxford E and S lesions.

### Association of SBP With Kidney Disease Progression

**Figure 1:**
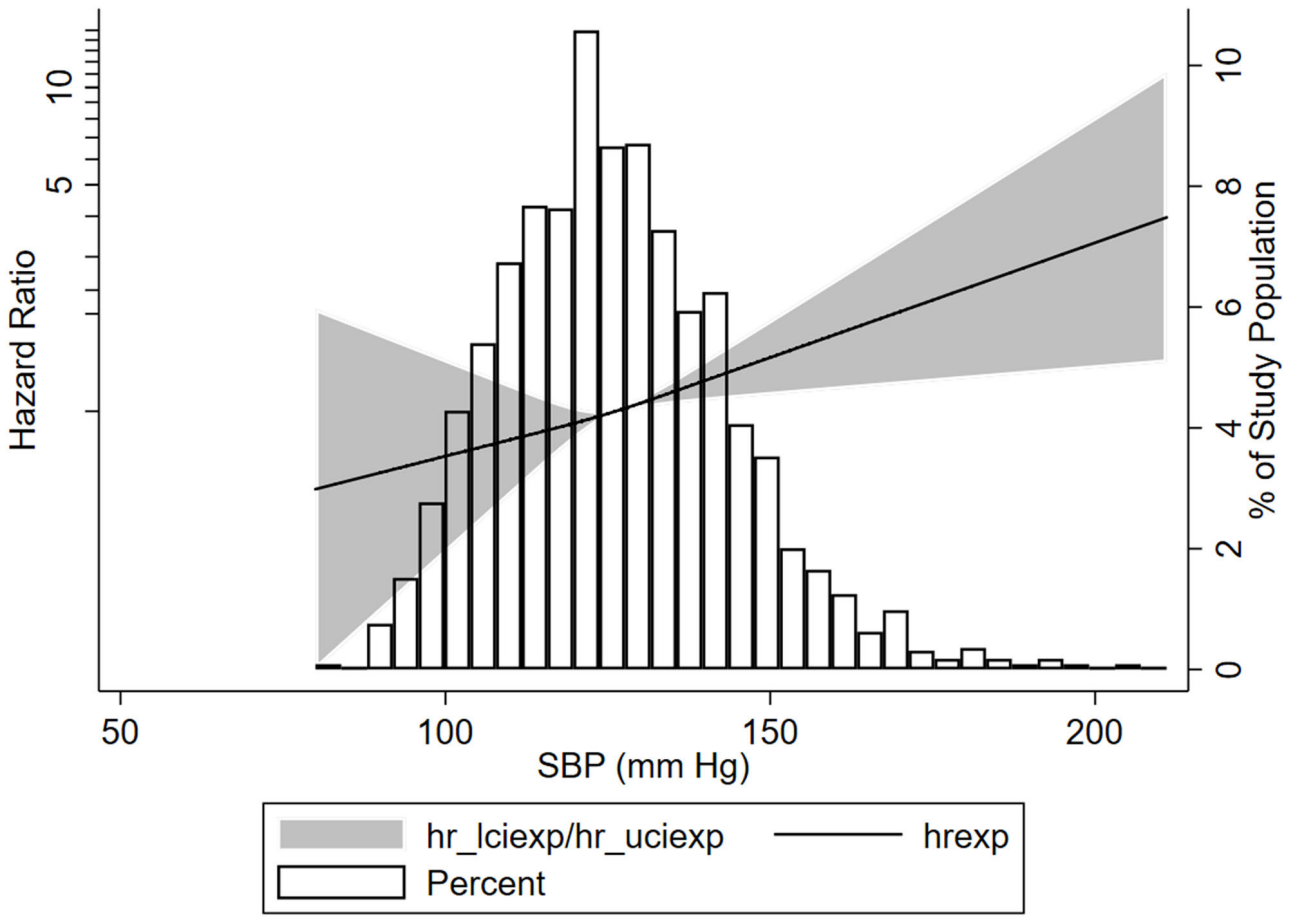
Association of systolic blood pressure (SBP) with the composite kidney failure events in patients with IgA nephropathy. Three knots at the 25th, 50th, and 75th percentiles of SBP levels were used to model the association between SBP levels and composite kidney failure events using restricted cubic splines, Composite kidney failure event was defined as 50% estimated glomerular filtration rate (eGFR) decline or end-stage kidney disease (ESKD). The solid line represents the estimated HR, the shaded area represents the 95% CI, the histogram represents the distribution of SBP levels. The models were adjusted for sex, age, baseline proteinuria, eGFR, antihypertension medications, and Oxford classification.

**Figure 2:**
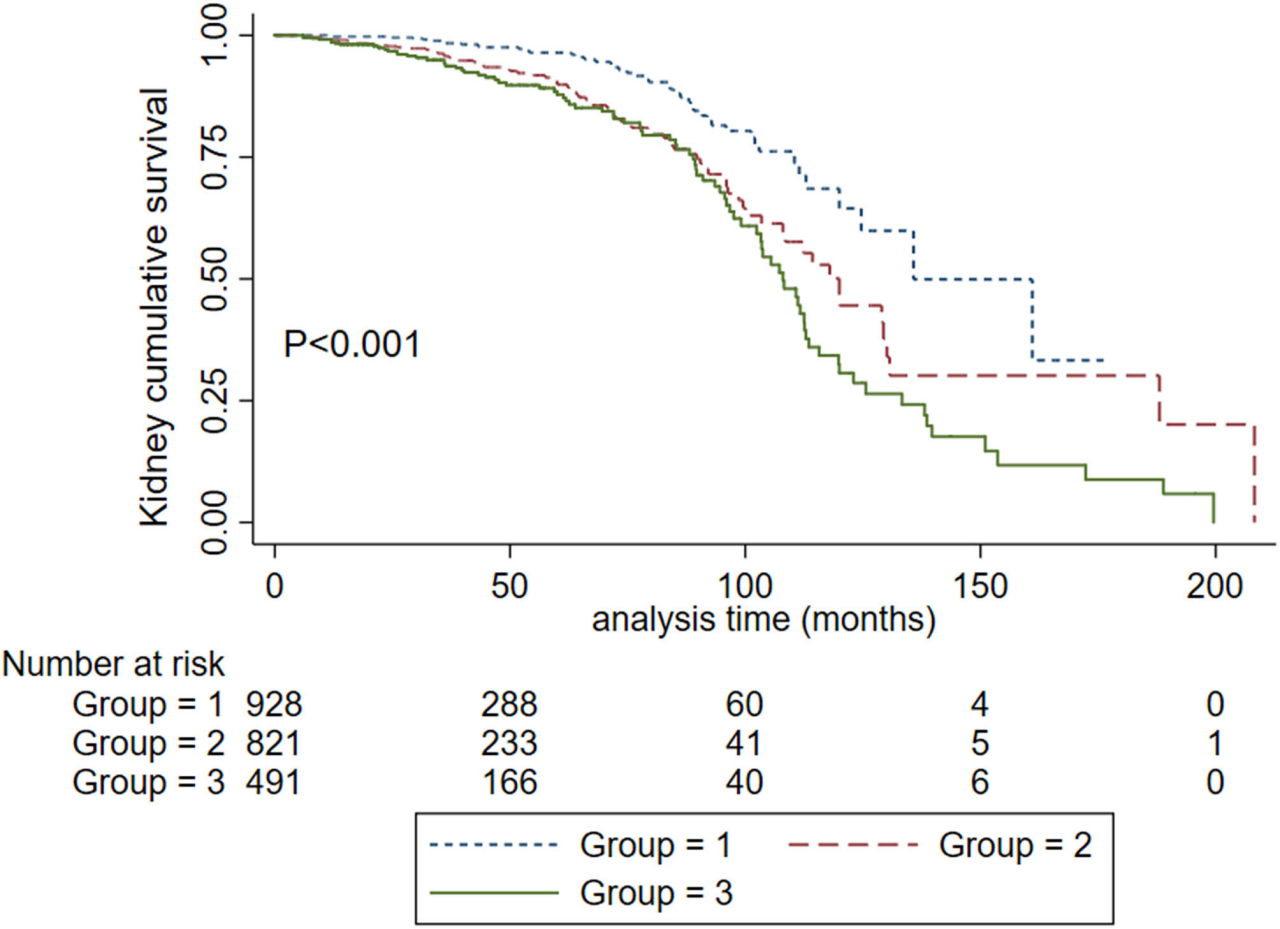
Kaplan-Meier renal survival curves in IgA nephropathy patients according to SBP levels. Group 1. Systolic blood pressure <120 mm Hg. Group 2, systolic blood pressure <140 mm Hg and ≥ 120 mm Hg. Group 3. Systolic blood pressure ≥ 140 mm Hg.

To obtain the relationship between SBP and the composite kidney failure events in IgA nephropathy, we firstly modeled SBP levels as a continuous variable using restricted cubic splines. A linear association was observed, with the higher SBP levels, the risk of kidney failure events was greater (Figure 1). We further used Cox models to evaluate the association between SBP and kidney failure events and found that ln-transformed SBP was an independent risk factor for kidney failure events (hazard ratio [HR] 7.09, [95% CI 1.86–27.03]; *P* = 0.004) after being adjusted for age, sex, initial proteinuria, eGFR, Oxford classificatio,n and the use of antihypertension agents (Table 2). In reference to Group 1 (SBP <120 mm Hg), the risk of composite kidney failure events was higher in patients with higher SBP levels: the hazard ratio was 1.85 (95% CI, 1.16–2.97) for Group 2 (SBP, 120–139 mm Hg), 1.87 (95%CI, 1.12–3.13) for Group 3 (SBP ≥140 mm Hg) (Table 2). As shown in Figure 2, renal survival deteriorated with the higher SBP levels, patients with SBP <120 mm Hg had a favorable survival compared to those with SBP <140 mm Hg (Figure 2).

**Table 2:** Risks of systolic blood pressure (SBP) in composite kidney disease progression in IgA nephropathy (IgAN).

**Groups**		**Hazard ration, 95% confidence Interval and** ***P*** **value**		
	**Unadjusted**	**Model 1**	**Model 2**	**Model 3**
The composite kidney failure event according to lnSBP levels	12.73 (5.36–30.23) <0.001	12.55 (5.28–29.82) <0.001	6.20 (1.98–19.41) 0.002	7.09 (1.86–27.03) 0.004
Group 1(SBP <120 mm Hg)	1 (Reference)	1 (Reference)	1 (Reference)	1 (Reference)
Group 2 (SBP, 120 to 139 mm Hg)	2.05 (1.43–2.94) <0.001	2.05 (1.42–2.95) <0.001	1.55 (1.04–2.32) 0.031	1.85 (1.16–2.97) 0.010
Group 3 (SBP≥140 mm Hg)	2.69 (1.87–3.85) <0.001	2.68 (1.86–3.85) <0.001	1.74 (1.15–2.65) 0.009	1.87 (1.12–3.13) 0.016
	<0.001	<0.001	0.010	0.015

*Composite kidney failure event was defined as 50% decline of eGFR or ESKD*.

*Model 1 was adjusted for age and sex; sex was expressed as a dichotomous variable*.

*Model 2 was adjusted for covariates in model 1 plus log-transformed proteinuria, eGFR and the use of anti-hypertension medications, the use of antihypertension expressed as a dichotomous variable (yes or no)*.

*Model 3 was adjusted for covariates in model 2 plus Oxford classification (MEST scores)*.

### Association Between SBP and Proteinuria

**Figure 3:**
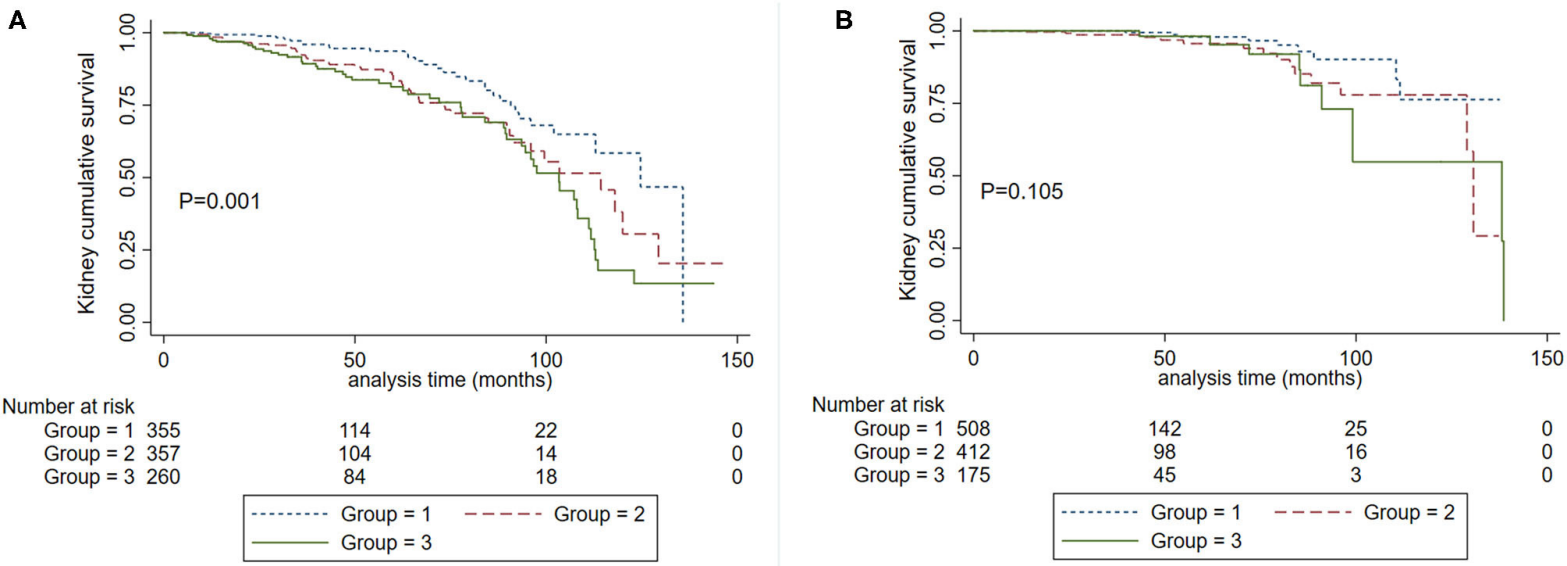
Kaplan-Meier renal survival curves according to SBP levels in patients with baseline proteinuria >1.0 g/d **(A)** and proteinuria ≤ 1.0 g/d **(B)**. Group 1. Systolic blood pressure <120 mm Hg. Group 2, systolic blood pressure <140 mm Hg and ≥120 mm Hg. Group 3. Systolic blood pressure ≥140 mm Hg.

We further evaluated the graded effect of SBP on renal outcomes in patients with proteinuria >1 g/d and ≤ 1 g/d. A total of 135 (13.89%) patients in the proteinuria >1 g/d group and 33 (3.14%) patients in proteinuria ≤ 1 g/d group developed composite kidney failure events (*P* < 0.001). In patients with proteinuria ≤ 1 g/d, SBP 120–139 mm Hg did not increase the risk for the composite kidney failure events compared to SBP <120 mm Hg (HR, 2.28; 95% CI, 0.997–5.222, *P* = 0.051). In those with proteinuria >1 g/d, SBP120-139 mm Hg was a risk factor for composite kidney failure events in reference to SBP <120 mm Hg (HR, 1.765, 95% CI, 1.129–2.760, *P* = 0.013). As shown in Figure 3, among patients with proteinuria >1 g/d, patients in SBP <120 mm Hg had a favorable prognosis compared with SBP <140 mm Hg (Figure 3A). However, no significant differences were observed in patients with SBP <120 mm Hg and SBP <140 mm Hg in the subgroup with proteinuria ≤ 1 g/d (Figure 3B). Then, we performed an interaction analysis and found a statistically significant multiplicative interaction for kidney failure events between proteinuria and SBP levels (*P* for interaction < 0.001).

### Association Between SBP and EGFR

**Figure 4:**
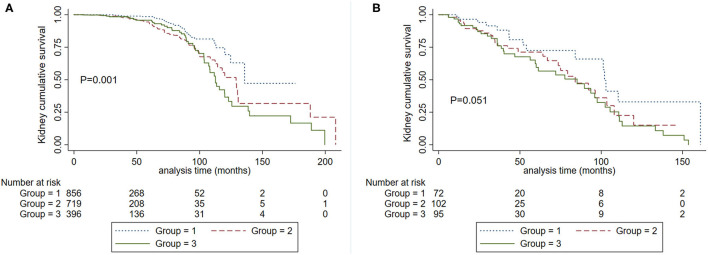
Kaplan-Meier renal survival curves according to SBP levels in patients with CKD 1-3a stage **(A)** and CKD 3b-4 stage **(B)**. Group 1. Systolic blood pressure <120 mm Hg. Group 2, systolic blood pressure <140 mm Hg and ≥120 mm Hg. Group 3. Systolic blood pressure ≥140 mm Hg.

We also analyzed the roles of eGFR and SBP on kidney disease progression. Patients were divided into two groups according to eGFR at the time of kidney biopsy: CKD1-3a stage and CKD 3b-4 stage. We firstly analyzed the graded effect of SBP on composite kidney failure events and found that after adjustment for age, sex, proteinuria, Oxford classification, and antihypertension medications, higher levels of SBP were independently associated with composite kidney failure events in patients with CKD1-3a stage, in reference to SBP <120 mm Hg, the hazard ratios were 1.88 (95% CI, 1.06–3.33, *P* = 0.031) for SBP between 120 and 139, and 2.29 (95% CI, 1.19–4.41, *P* = 0.013) for SBP ≥140 mm Hg. For patients with CKD 3b-4 stage, higher levels of SBP were not associated with poor renal outcome, the hazard ratios were 0.72 (95% CI, 0.23–2.23, *P* = 0.566) for SBP between 120 and 139, and 0.96 (95% CI, 0.30–3.08, *P* = 0.951) for SBP ≥140 mm Hg. These results were similar in cumulative kidney survival curves (Figure 4) in patients with CKD 1-3a stage, those with SBP <120 mm Hg compared to <140 mm Hg had improved kidney survival (Figure 4A). In cases with CKD 3b-4 stage, those with SBP <120 mm Hg had a similar renal survival compared to SBP <140 mm Hg (Figure 4B).

### Association Between SBP and Age

**Figure 5:**
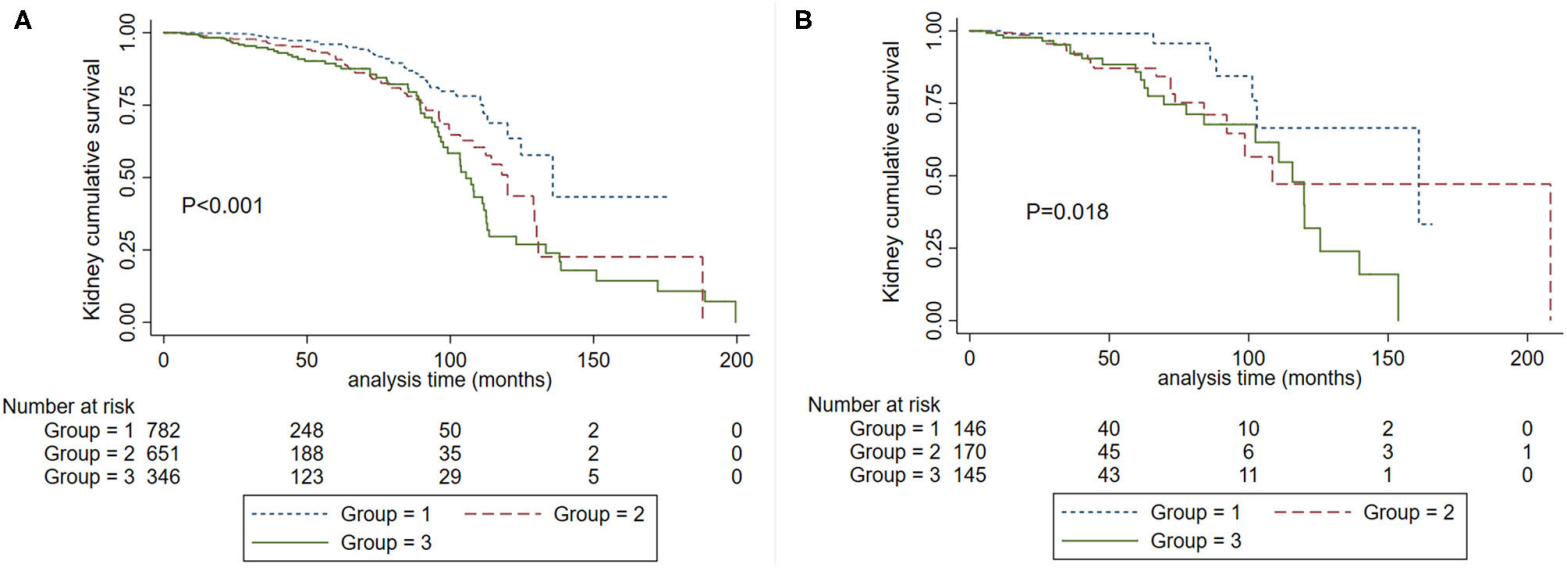
Kaplan-Meier renal survival curves according to SBP levels in patients with age <50 years **(A)** and ≥ 50 years **(B)**. Group 1. Systolic blood pressure <120 mm Hg. Group 2, systolic blood pressure <140 mm Hg and ≥120 mm Hg. Group 3. Systolic blood pressure ≥140 mm Hg.

Cumulative kidney survival curves demonstrated that patients with SBP <120 mm Hg had a favorable prognosis compared to SBP 120–139 mm Hg and SBP ≥140 mm Hg either in the subgroup with age <50 years (*P* < 0.001) or age ≥50 years (*P* = 0.018) (Figure 5). In the proportional hazard ratio model, we found that in reference to SBP <120 mm Hg, the hazard ratios were 1.87 (95% CI, 1.12–3.13, *P* = 0.017) for SBP between 120 and 139 and 1.92 (95% CI, 1.10–3.36, *P* = 0.023) for SBP ≥ 140 mm Hg in patients with years in age <50. In the subgroup with years in age ≥ 50, the hazard ratios were 1.72 (95% CI, 0.43–6.85, *P* = 0.440) for SBP between 120 and 139, and 2.27 (95% CI, 0.59–8.79, *P* = 0.235) for SBP ≥140 mm Hg.

### Association of Treatment of Antihypertension With Kidney Disease Progression

**Figure 6:**
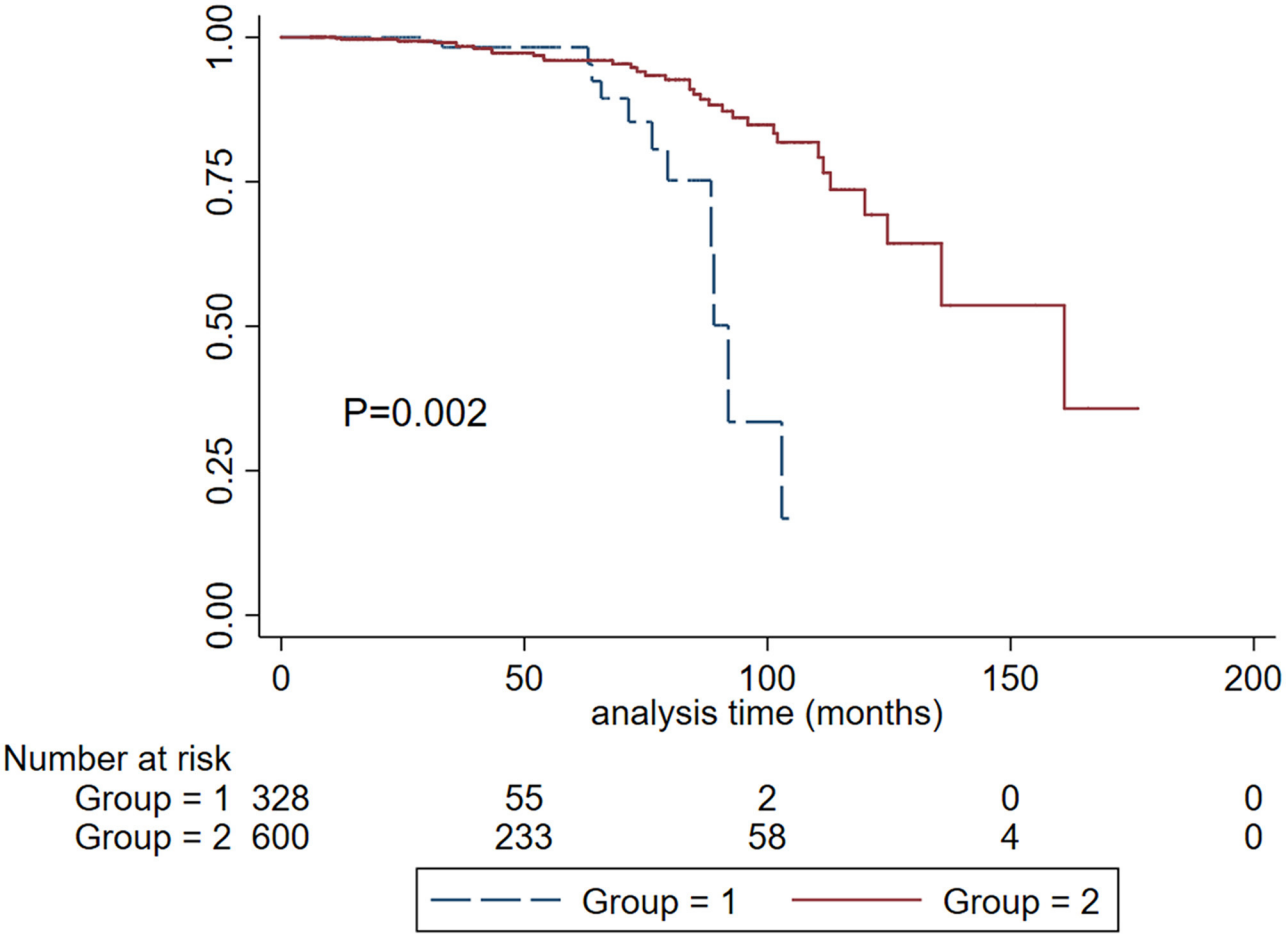
Kaplan-Meier renal survival curves according to the treatment of antihypertension in patients with SBP <120 mm Hg. Group 1. Without antihypertension treatment. Group 2, with antihypertension treatment.

In patients with SBP <120 mm Hg, there were 600 (64%) cases that received antihypertension treatment. Treatment with antihypertension was a protective factor in the unadjusted Cox model (HR, 0.341; 95% CI, 0.165–0.702; *P* = 0.004) after being adjusted for traditional risk factors, treatment with antihypertension was significantly associated with reduced risk for the composite event (HR, 0.329; 95% CI, 0.121–0.899; *P* = 0.030). Kidney cumulative survival analysis demonstrated that patients with antihypertension treatment had a favorable renal survival compared to those without antihypertension treatment (Figure 6).

## Discussion

The KDIGO 2021 Clinical Practice Guideline proposes an SBP target of <120 mm Hg for patients with CKD not receiving dialysis because of the cardiovascular and survival benefits of the lower SBP target. Whether lowering SBP from <140 mm Hg to <120 mm Hg is renoprotective is uncertain, especially in those with advanced CKD, proteinuria > 1 g/d, and the young cases with age <50 years (13, 14). In this cohort study with more than 2,000 patients with IgA nephropathy and 217 composite kidney failure events, we demonstrated that SBP <120 mm Hg was associated with reduced incidence of composite kidney failure events compared to SBP <140 mm Hg, especially in those with proteinuria >1g/d, CKD 1-3a stage and age <50 years. These findings suggest the potential renoprotective benefits of therapy targeting SBP <120 mm Hg in IgA nephropathy patients not receiving dialysis.

The effect of lowering SBP on the progression of kidney disease was less certain. In earlier RCTs studies, more vs. less intensive BP lowering did not demonstrate a benefit of lowering BP targets on kidney failure events in CKD populations (15–17). However, further analyses of these and other studies have established that lowering SBP reduced the rates of CKD progression among patients with greater proteinuria (18–20, 24). Some studies also demonstrated a log-liner relationship between blood pressure and the risk of myocardial infarction, heart failure, stroke, and other cardiovascular events rather than renal failure events (25). Lowering blood pressure could significantly reduce cardiovascular disease and mortality (26, 27). However, the extent to which blood pressure can be safely reduced has not yet been determined. Regarding optimal blood pressure management, recommendations from different guidelines are also different. Largely based on cardioprotective, survival, and potential cognitive benefits, the KDIGO 2012 BP guideline recommended and SBP <130/80 mm Hg (28). The 2017 ACC/AHA BP guideline suggested a target of <130/80 mm Hg for patients with CKD, which was more aggressive than that recommended by the European Society of Hypertension (ESH) (SBP target 130–139 mm Hg), the European Society of Cardiology (ESC), and the National Institute of Health and Care Excellence (NICE) recommended SBP target of 120–139 mm Hg (29–32). Recently published KDIGO guidelines proposed an SBP target <120 mm Hg which is consistent with Hypertension Canada recommendation partly based on the results of the Systolic Blood Pressure Intervention Trial (SPRINT) results, which show cardiovascular and survival benefits rather than renoprotective benefits (33, 34).

It is established that SBP-lowering is likely renoprotective based on lowering SBP from >160 mm Hg to <140 mm Hg (13). Whether lowering SBP from <140 mm Hg to <120 mm Hg has a renoprotective effect is unknown. In the SPRINT study, after a median follow-up of 3.3 years, 15 patients (1.1%) with CKD in SBP <120 mm Hg group and 16 (1.2%) participants in SBP <140 mm Hg group developed kidney outcome defined as 50% eGFR decline or ESKD (HR, 0.9; 95%CI, 0.44–1.83), the similar incidence of kidney outcome between two groups may be due to the small numbers of events (35). In the current IgA nephropathy cohort with 2,240 participants and 46 (4.96%) patients in SBP <120 mm Hg and 87 (17.72%) patients reached composite kidney failure events, we found that compared to SBP <140 mm Hg, SBP <120 mm Hg reduced the incidence of composite kidney failure events, the association was independent of established risk factors including initial proteinuria, eGFR, and Oxford classification (MEST-C scores). Importantly, we established that the effect of SBP on kidney failure events had an interaction with initial proteinuria, being much greater in patients with proteinuria >1.0 g/d. For patients with proteinuria >1.0 g/d, SBP <120 mmHg decreased the kidney failure events compared with SBP <140 mm Hg by 76.5%. For those with proteinuria ≤ 1.0 g/d, lowering SBP from <140 mm Hg to <120 mm Hg did not improve kidney outcomes. This result is similar to prior study findings that in patients with greater proteinuria, intensive blood pressure lowering reduces the kidney failure events (18–20, 24). These results highlight the importance of lowering SBP from <140 mm Hg to <120 mm Hg during the therapy in IgA nephropathy patients, especially in those with proteinuria >1.0 g/d.

Another key finding from this study was that in patients with CKD 1-3a stage rather than CKD 3b-4 stage, SBP <120 mm Hg was associated with favorable kidney outcomes compared to SBP <140 mm Hg. This finding is similar to those from the ATACH-2 trial, in which the odds of death or disability were significantly higher in intensive BP-lowering therapy patients with CKD, however, the kidney outcome was not assessed in the study (36). Given these findings, lowering SBP <120 mm Hg should be made, especially in those with CKD stage 1-3a.

The strengths of this study include the large sample sizes and a large number of kidney failure events, which provided good study power to assess the association of SBP lowering with hard endpoints. We found that compared to SBP <140 mm Hg, SBP <120 mm Hg reduced the kidney failure events in patients with IgA nephropathy, the association was independent of the Oxford classification and other traditional clinical risk factors. This study also has some limitations: First, all patients were from a single-center study with a relatively short follow-up time, hence, the findings need to be confirmed in other populations. Second, in our study, >95% of patients received steroids, renin-angiotensin-aldosterone system inhibitors (RAASis) during follow-up, and the treatment was similar in three groups, therefore, we did not include the use of RAASi and immunosuppressives into the analysis.

In conclusion, in this large cohort study, we demonstrated that in patients with IgA nephropathy, SBP was independently associated with kidney failure events, lowering SBP from <140– <120 mm Hg could improve the renal prognosis, the effect was greater in those with baseline proteinuria > 1.0 g/d, CKD 1-3a stage and age <50 years, for those with proteinuria ≤ 1.0 g/d and CKD 3b-4 stage, the renoprotective was not significant.

## Data Availability Statement

The original contributions presented in the study are included in the article/supplementary material, further inquiries can be directed to the corresponding author.

## Ethics Statement

All procedures performed in this study protocol were reviewed and approved by the Ethics Committee of the institute of the First Affiliated Hospital, College of Medicine, Zhejiang University.

## Author Contributions

GY and JC: research idea and study design. GY, JC, and YJ: data acquisition. GY: data analysis, interpretation, and statistical analysis. HL, XL, and JC: supervision or mentorship. All authors approved the final version of the manuscript. All authors contributed to the article and approved the submitted version.

## Funding

This work was supported by grants LQ19H050009 and LY19H050007 from the Zhejiang Provincial Natural Science Foundation of China and grant 2016KYA087 from the Zhejiang Medical and Health Science and Technology Project.

## Conflict of Interest

The authors declare that the research was conducted in the absence of any commercial or financial relationships that could be construed as a potential conflict of interest.

## Publisher's Note

All claims expressed in this article are solely those of the authors and do not necessarily represent those of their affiliated organizations, or those of the publisher, the editors and the reviewers. Any product that may be evaluated in this article, or claim that may be made by its manufacturer, is not guaranteed or endorsed by the publisher.
